# Perforated Kirschner wire tension band in the treatment of Mayo IIA olecranon fractures

**DOI:** 10.3389/fsurg.2024.1500317

**Published:** 2024-11-26

**Authors:** Xiang Yu, Qi Li, Yu-Zhi Li, Hai-Jian Lu, Rong-Guang Ao, Bing-Li Liu

**Affiliations:** Department of Orthopedic Surgery, Shanghai Seventh People’s Hospital, Shanghai, China

**Keywords:** olecranon fracture, kirschner wire tension band, olecranon anatomical plate, fracture internal fixation, surgical treatment

## Abstract

**Purpose:**

To explore the clinical efficacy of perforated Kirschner wire tension band in the treatment of Mayo IIA olecranon fracture.

**Method:**

A retrospective study was conducted to analyze the clinical data of 96 adult patients with olecranon fractures of the ulna. Thirty-four cases underwent perforated Kirschner wire tension band fixation(group A), which included 21 males and 13 females, with an average age of 49.1 ± 11.57 years. Thirty-two cases received fixation with an olecranon anatomical plate (group B), comprising 19 males and 13 females, with an average age of 48.9 ± 8.84 years. Additionally, 30 cases were treated with ordinary Kirschner wire tension band fixation (group C), consisting of 18 males and 12 females, with an average age of 46.6 ± 12.03 years. The study compared various outcomes among the three groups, including operation time, intraoperative blood loss, number of fluoroscopy exposures, postoperative visual analogue score (VAS), fracture healing time, internal fixation failure rates, skin irritation rates, and the Broberg-Morrey score for elbow joint function at the final follow-up.

**Result:**

All patients were followed for a duration of 15 to 21 months, with an average follow-up period of 18 months. The operation time, intraoperative blood loss, number of fluoroscopy sessions, fracture healing time, and incidence of skin irritation for group A were recorded as (73.8 ± 11.72) min, (113.5 ± 20.73) milliliters, (7.5 ± 1.96) times, and (3.7 ± 1.46) months, respectively, with 1 case of skin irritation. In group B, the corresponding values were (98.4 ± 10.46) min, (154.7 ± 20.11) milliliters, (11.8 ± 2.78) times, and (4.3 ± 1.69) months, with 5 cases of skin irritation. For group C, the values were (81.7 ± 15.66) min, (115.5 ± 18.82) milliliters, (7.3 ± 1.99) times, and (4.3 ± 1.86) months, with 7 cases of skin irritation. Group A demonstrated superior outcomes compared to group B in terms of intraoperative blood loss and number of fluoroscopy sessions, and outperformed both group B and group C regarding operation time, fracture healing time, and skin irritation. These differences were statistically significant (*P* < 0.05).

**Conclusion:**

Perforated Kirschner wire Tension band is an effective method for treating olecranon fractures. This technique is associated with a short operative time, minimal intraoperative blood loss, and a reduced need for fluoroscopy. Additionally, it promotes fracture healing and significantly reduces the risk of complications, such as postoperative skin irritation.

## 1 Introduction

Olecranon fractures are prevalent in adult upper limbs, comprising 10% of all upper limb fractures. Among these, Mayo IIA fractures are the most common, accounting for 73.5% of olecranon fractures (1). Current treatment principles emphasize anatomical alignment, secure fixation, and early functional rehabilitation (2). Surgical intervention not only reduces the incidence of traumatic arthritis but also facilitates satisfactory elbow joint function. Common surgical internal fixation techniques include Kirschner wire tension band and anatomical steel plate. However, conventional Kirschner wire tension band can lead to issues such as skin irritation and loosening of internal fixation, while anatomical plates may result in increased surgical trauma (3). The perforated Kirschner wire represents an advancement over standard Kirschner wire (4). Nevertheless, there is a paucity of applications and comparative studies regarding ulnar olecranon fractures. In this study, we conducted a retrospective analysis involving 96 patients with olecranon fractures to evaluate the clinical efficacy of perforated Kirschner wire tension band in the treatment of Mayo IIA fractures of the olecranon.

## 2 Material and methods

### 2.1 Study subjects and design

The study was conducted at Shanghai Seventh People's Hospital. A total of 96 patients with olecranon fracture of ulna, admitted to our hospital, were assigned to receive open reduction and internal fixation (ORIF). All patients were diagnosed with olecranon fractures using anteroposterior and lateral x-rays of the elbow joint. There were no established criteria for selecting indications for various internal fixation methods; thus, the decision largely relies on the surgeon's experience. Among the patients, 34 received perforated Kirschner wire tension band fixation (Group A), 32 underwent olecranon anatomical plate fixation (Group B), and 30 were treated with standard K-wire tension band fixation (Group C). Before enrollment, subjects were informed about the study protocol, Informed consent was obtained from all patients. All procedures in this study adhered to the ethical principles of clinical research outlined in the Declaration of Helsinki and received approval from the Medical Ethics Committee of Shanghai Seventh People's Hospital (SSJW-2020045).

### 2.2 Inclusion and exclusion criteria

Inclusion criteria: (1) diagnosis of Mayo IIA olecranon fracture, (2) presence of a fresh closed fracture, (3) age between 18 and 65 years. Exclusion criteria: (1) presence of Mayo type I, Mayo type IIB, or Mayo type III fractures, (2) concurrent fractures in other parts of the ipsilateral limb, (3) associated neurovascular injury, (4) involvement of significant organ damage, (5) presence of ipsilateral elbow joint deformity due to congenital or acquired conditions, (6) pathological fractures.

### 2.3 Intervention measures

#### 2.3.1 Surgical Method

**Figure 1:**
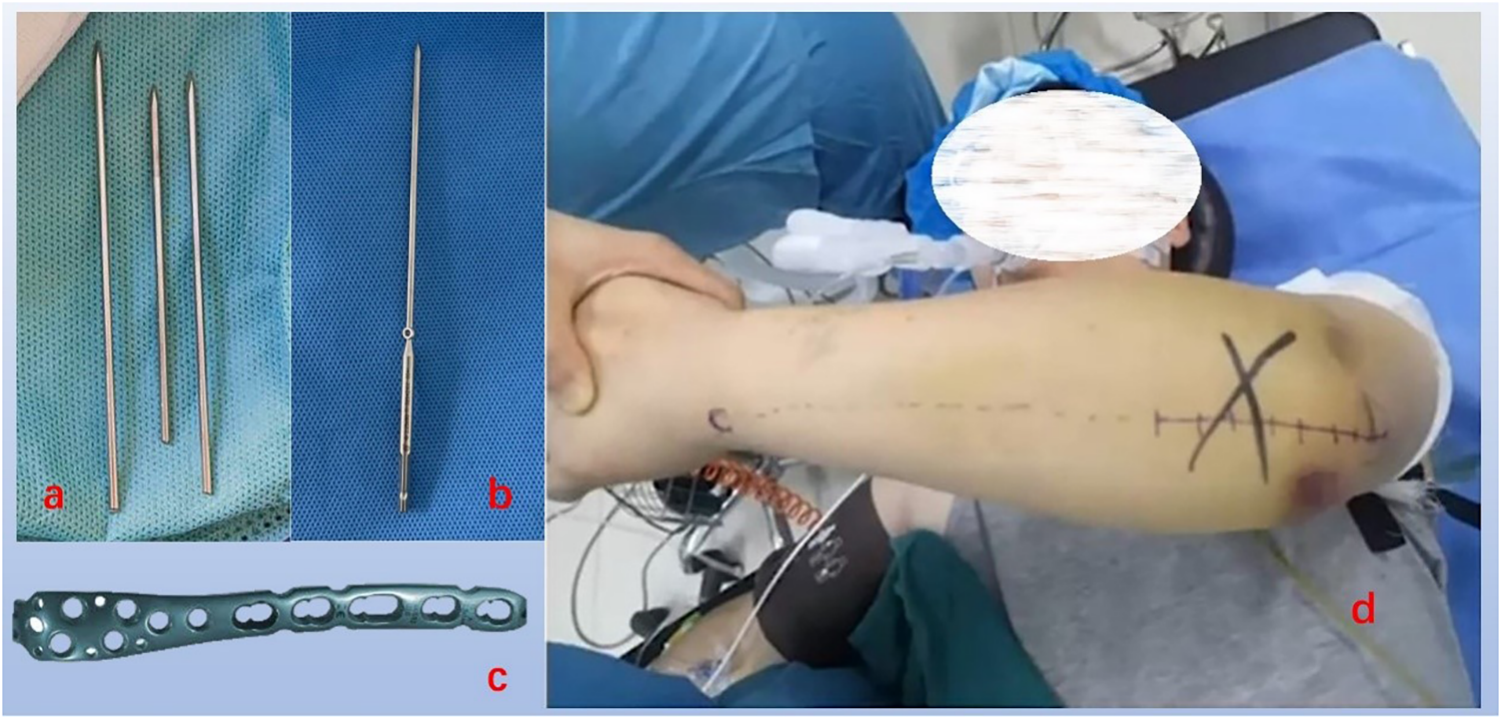
Internal fixation instruments and surgical incision, **(a)** ordinary Kirschner wire, **(b)** perforated Kirschner wire, **(c)** olecranon plate, **(d)** surgical position and surgical incision.

The patient is positioned supine (5). A pneumatic tourniquet is applied to the upper arm, with the pressure set at 220 mmHg. Following the administration of brachial plexus anesthesia, routine disinfection and draping are conducted. An arc-shaped incision is made behind the elbow joint, centered on the fractured end of the olecranon (Figure 1d). After incising layer by layer, the fracture's broken end is fully exposed, and any soft tissue and small bone debris within the joint and at the fracture site are removed. The elbow joint is then slightly flexed to facilitate the operation and reduction.

#### 2.3.2 Group A

After reduction, fracture was maintained by the forceps, and two Kirschner wires (Figure 1b) with a diameter of 2.0 mm were inserted from the proximal end of the olecranon. The two Kirschner wires were placed parallel within the ulnar medullary cavity (6). Following confirmation of satisfactory fracture reduction and Kirschner wire positioning via the C-arm x-ray machine, a hole was drilled 3 cm distal to the fracture line, perpendicular to the longitudinal axis of the ulna, with a diameter of approximately 2 mm. The same steel was inserted into both the anchor hole and the Kirschner wire hole, with the cable crossed in an “8” shape on the dorsal side of the olecranon. A hammer was used to tap the pinhole at the end of the Kirschner wire into the starting part of the triceps brachii muscle. The cable was then tightened. Fluoroscopy with the C-arm x-ray machine reconfirmed the fracture reduction, and upon satisfaction with the Kirschner wire positioning, the cable lock was clamped, and the pre-broken part of the K-wire tail was severed (Figure 1).

#### 2.3.3 Group B

Following temporary fixation with Kirschner wires and confirmation of fracture reduction using C-arm x-ray fluoroscopy, an olecranon anatomical plate (Figure 1c) of appropriate length was positioned on the posterior aspect of the olecranon of the ulna. Subsequently, 2 to 3 cancellous plates were placed proximal to the fracture site. Bone screws were then inserted, ensuring the appropriate number of bicortical screws was utilized at the distal end of the fracture (7).

#### 2.3.4 Group C

Group C employs a surgical method identical to that of Group A. When working with the standard Kirschner wire (Figure 1a), bend the needle tail, trim it to a short length, and use a hammer to gently tap the bent section into the origin of the triceps brachii muscle.

After confirming through direct observation that the passive movement of the elbow joint is satisfactory and that the internal fixation is stable, the wound is irrigated with normal saline and closed layer by layer with sutures.

#### 2.3.5 Postoperative Treatment

Routine cleaning and care of the postoperative wound should be conducted without the application of plaster or braces. Passive functional exercises were initiated on the third day following the operation, while active functional exercises commenced two weeks post-surgery and were gradually intensified (8).

### 2.4 Outcome measures

#### 2.4.1 The operation time

The operation time starts from skin incision to the end of skin suturing.

#### 2.4.2 Intraoperative blood loss

Blood loss was quantified by measuring the volume of blood collected in the bucket of the negative pressure aspirator at the conclusion of the operation.

#### 2.4.3 Number of fluoroscopy sessions

Record the number of fluoroscopy performed during surgery.

#### 2.4.4 Postoperative visual analogue score (VAS)

The mean VAS value from the first day to one week post-surgery was utilized as the postoperative pain score.

#### 2.4.5 Fracture healing time and internal fixation failure

Standard frontal and lateral x-rays were obtained on the second day, two weeks, and once a month following surgery to monitor complications such as internal fixation failure. All patients were monitored by the regular physician, who reviewed their x-rays. His criteria for assessing fracture healing primarily rely on clinical examinations and imaging findings. At the final follow-up, x-ray films were reviewed to assess bone healing prior to the removal of internal fixation.

#### 2.4.6 Skin irritation

The primary criteria for assessing skin irritation include: (1). The patient's primary complaint regarding irritation or pressure on the subcutaneous tissue caused by the internal fixation of the elbow joint. (2). Palpation; when the patient's elbow joint is pressed, the internal fixation may be palpable, potentially eliciting pain. (3). Visual inspection; the local skin in the area of the internal fixation at the elbow joint may appear bulging, red, and swollen to the naked eye.

#### 2.4.7 Elbow joint function

The Broberg-Morrey score for elbow joint function was evaluated at the last follow-up based on four criteria: movement, strength, stability, and pain, with a maximum total score of 100 points.

### 2.5 Statistical analysis

Statistical analysis was conducted using SPSS version 23.0. Data are presented as mean ± standard deviation. For normally distributed data, one-way analysis of variance (ANOVA) was employed to compare the three groups. In cases where the data were not normally distributed, the non-parametric Kruskal-Wallis rank sum test was utilized. For items exhibiting differences between groups, additional multiple comparisons were performed. When the multiple comparison data indicated homogeneous variances, the Least Significant Difference (LSD) test was applied; conversely, when the variances were unequal, Tamhane's *T*2-test was used. Count data were analyzed using either the chi-square test or Fisher's exact test. A *P* value of less than 0.05 was considered statistically significant.

## 3 Results

### 3.1 Baseline patient profiles

A total of 96 adult patients with ulnar olecranon fractures were included in the study, comprising 58 males and 38 females, with ages ranging from 35 to 60 years (mean age: 48.2 ± 11.7 years). Among these, 34 patients underwent perforated Kirschner wire tension band fixation (group A), which included 21 males and 13 females, with a mean age of 49.1 ± 11.57 years. The causes of injury in this group were as follows: 10 cases resulted from traffic accidents, 8 cases were due to falls from a height, and 16 cases were attributed to falls on the ground. The mean time from injury to surgery was 2.2 ± 1.03 days. In group B, which consisted of 32 patients receiving olecranon anatomical plate fixation, there were 19 males and 13 females, with a mean age of 48.9 ± 8.84 years. The causes of injury in this group included 7 traffic accidents, 12 falls from a height, and 13 falls on the ground, with a mean time from injury to surgery of 2.4 ± 1.24 days. Group C included 30 patients who received ordinary K-wire tension band fixation, composed of 18 males and 12 females, with a mean age of 46.6 ± 12.03 years. The causes of injury in this group were 6 traffic accidents, 11 falls from a height, and 13 falls on the ground, with a mean time from injury to surgery of 2.2 ± 1.04 days. There were no statistically significant differences in the demographic and clinical characteristics among the three groups, including gender, age, cause of injury, and time from injury to surgery (*P* > 0.05). Patients' clinical data are presented in Table 1.

**Table 1:** Comparison of general information between three groups of patients with olecranon fracture.

Group	Number	Gender		Age (mean ± SD)	Cause of injury			Time from injury to surgery (day, mean ± SD)
		Male	Female		Traffic injury	Fall from height	Fall from flat surface	
Group A	34	21	13	49.1 ± 11.57	10	8	16	2.2 ± 1.03
Group B	32	19	13	48.9 ± 8.84	7	12	13	2.4 ± 1.24
Group C	30	18	12	46.6 ± 12.03	6	11	13	2.2 ± 1.04
*X^2^/F*		0.043		0.503	2.089			0.380
*P*		0.979		0.606	0.719			0.685

Group A is the perforated Kirschner wire tension band group, Group B is the olecranon anatomical plate group and Group C is normal Kirschner wire tension band group.

### 3.2 Clinical efficacy

**Figure 2:**
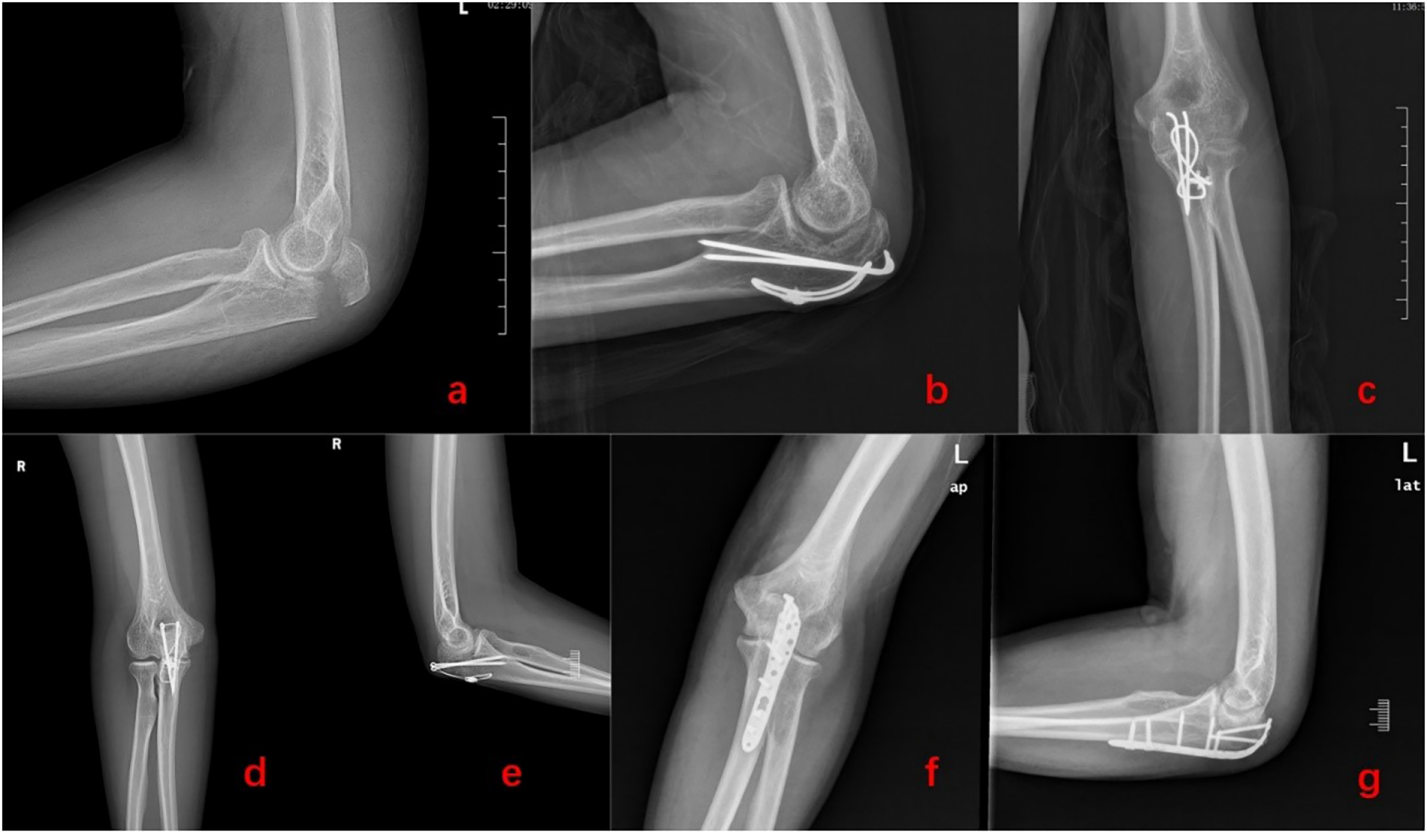
Internal fixation of olecranon fracture, **(a)** lateral x-ray showing olecranon fracture, **(b)** lateral x-ray after ordinary Kirschner wire internal fixation, **(c)** anteroposterior x-ray after ordinary Kirschner wire internal fixation, **(d)** anteroposterior x-ray after perforated Kirschner wire internal fixation, **(e)** lateral x-ray after perforated Kirschner wire internal fixation, **(f)** anteroposterior x-ray after internal fixation of olecranon plate, **(g)** lateral x-ray after internal fixation of olecranon plate.

All patients were followed for a duration of 15 to 21 months, with an average follow-up of 18 months, and all fractures successfully healed. Typical cases are illustrated in Figure 2. For patients experiencing skin irritation, it is recommended to rest, immobilize the affected area, and apply Voltaren ointment externally. Internal fixation failure was observed in 2 patients, both of whom experienced loosening of Kirschner wires without any significant fracture displacement. The fractures subsequently healed normally following plaster fixation.

In Group A, the operation time, intraoperative blood loss, number of fluoroscopy sessions, postoperative VAS score, fracture healing time, internal fixation failure, skin irritation, and Broberg-Morrey score were recorded as (73.8 ± 11.72) min, (113.5 ± 20.73) ml, (7.5 ± 1.96) times, (5.0 ± 0.97) min, (3.7 ± 1.46) months, 0 cases, 1 case, and (90.8 ± 4.31) min, respectively. In Group B, the corresponding values were (98.4 ± 10.46) min, (154.7 ± 20.11) ml, (11.8 ± 2.78) times, (5.0 ± 1.05) min, (4.3 ± 1.69) months, 0 cases, 5 cases, and (90.1 ± 3.77) min. Group C exhibited values of (81.7 ± 15.66) min, (115.5 ± 18.82) ml, (7.3 ± 1.99) times, (4.87 ± 0.94) min, (4.3 ± 1.86) months, 2 cases, 7 cases, and (90.5 ± 4.13) min. The various outcome indicators are summarized in Table 2.

**Table 2:** Comparison between three groups (mean ± SD).

Item	Group A (*n* = 34)	Group B (*n* = 32)	Group C (*n* = 30)	*X^2^/F*	*P*
Operation time (min)	73.8 ± 11.72	98.4 ± 10.46	81.7 ± 15.66	77.630	0.000
Intraoperative blood loss (ml)	113.5 ± 20.73	154.7 ± 20.11	115.5 ± 18.82	174.783	0.000
Number of fluoroscopy	7.5 ± 1.96	11.8 ± 2.78	7.3 ± 1.99	124.594	0.000
visual analog score (VAS)	5.0 ± 0.97	5.0 ± 1.05	4.87 ± 0.94	0.241	0.785
Fracture healing time (months)	3.7 ± 1.46	4.3 ± 1.69	4.3 ± 1.86	8.788	0.000
Failure of internal fixation (number, %)	0 (0)	0 (0)	2 (6.67)	4.494	0.106
Skin irritation (number, %)	1 (2.94)	5 (15.6)	7 (23.3)	6.122	0.035
Broberg-Morrey score	90.8 ± 4.31	90.1 ± 3.77	90.5 ± 4.13	0.222	0.801

Group A is the perforated Kirschner wire tension band group, Group B is the olecranon anatomical plate group and Group C is normal Kirschner wire tension band group.

The comparison among the three groups revealed statistically significant differences in operation time, intraoperative blood loss, number of fluoroscopy images, fracture healing time, and skin irritation (*P* < 0.05). The results of these comparisons are presented in Table 2. Further multiple comparison analysis indicated that group A outperformed group B in terms of intraoperative blood loss and the number of fluoroscopy images, and also surpassed both group B and group C regarding operation time, fracture healing time, and skin irritation, with these differences being statistically significant (*P* < 0.05). The results of these comparisons are detailed in Table 3.

**Table 3:** Multiple comparison results between the perforated Kirschner wire tension band group and the other two groups.

Item			*P*
Operation time (min)	Group A	Group B	0.000
		Group C	0.000
Intraoperative blood loss (ml)	Group A	Group B	0.000
		Group C	0.431
Number of fluoroscopy	Group A	Group B	0.000
		Group C	0.540
Fracture healing time (months)	Group A	Group B	0.000
		Group C	0.001
Skin irritation (number,%)	Group A	Group B	0.041
		Group C	0.004

Group A is the perforated Kirschner wire tension band group, group B is the olecranon anatomical plate group and Group C is normal Kirschner wire tension band group.

## 4 Discussion

Olecranon fractures are prevalent in adult upper limbs, constituting 10% of all upper limb fractures (2). Among these, Mayo type IIA fractures are the most common, representing 73.5% of total olecranon fractures. As an intra-articular fracture, poor reduction of the joint surface can lead to unevenness, resulting in limited elbow joint movement and impaired function (9). Consequently, surgical intervention is necessary to achieve anatomical reduction and prevent the onset of traumatic arthritis (10). Currently, various internal fixation options are available for the surgical treatment of olecranon fractures (11). Traditional Kirschner wire tension bands are the most frequently utilized in clinical practice due to their minimal trauma and straightforward application (12, 13). However, these tension bands are also associated with complications such as internal fixation failure and skin irritation, which can compromise clinical outcomes. An alternative internal fixation method is the use of anatomical steel plates, which offer greater strength and a reduced incidence of fixation failure. Nevertheless, the application of anatomical plates necessitates extensive periosteal stripping, potentially resulting in significant damage to the blood supply of the fracture site (14). In this study, we explored the use of perforated Kirschner wire tension bands, commonly employed in patellar fractures, as a modified tension band for the olecranon of the ulna. We compared the clinical efficacy of this modified Kirschner wire tension band with that of anatomical plates and traditional Kirschner wires.

The results of this study indicate that the operation time, intraoperative blood loss, and number of radiographs required for the perforated Kirschner wire tension band are significantly less than those associated with the anatomical plate. Despite being an improved design over the traditional Kirschner wire, the surgical techniques for fracture end exposure, reduction, and internal fixation placement remain similar, thereby not increasing the complexity of the procedure and making it easier for surgeons to adopt (15). Conversely, the fixation process with the steel plate necessitates extensive periosteal stripping and involves multiple steps, including plate placement, hole rotation, depth measurement, and screw insertion. These factors contribute to increased operation time, greater intraoperative blood loss, and a higher number of fluoroscopy exposures (16). Furthermore, the fracture healing time in the perforated Kirschner wire tension band group averaged (3.7 ± 1.46) months, which is shorter than the (4.3 ± 1.69) months observed in the olecranon anatomical plate group. This suggests that the use of perforated Kirschner wire tension bands may facilitate fracture healing. The tightening of the “8” tension band during the procedure converts tension into pressure at the anchor and pinholes, enhancing contact between the fracture ends (17). Additionally, this method does not require extensive periosteal dissection (18), thereby better preserving the bone surface and minimizing blood loss.

Perforated Kirschner wire tension bands have been widely utilized in the treatment of patellar fractures. Their unique design addresses the issue of ordinary Kirschner wires, which are prone to withdrawal and can lead to failure of internal fixation (19). We applied this technique to olecranon fractures and observed comparable clinical outcomes. In this study, we noted two instances of internal fixation failure attributed to the withdrawal of standard Kirschner wires. Based on surgical experience, the use of perforated Kirschner wires for olecranon fractures allows the steel cable to pass through the needle holes at the tail end, effectively limiting the rotation and sliding of the Kirschner wire body within the bone. The presence of an anchor hole at the distal end of the fracture, along with the proximal pinhole and distal anchor hole, ensures secure fixation of the Kirschner wire through the “8” shaped steel cable. This configuration forms a complete and mutually restrained closed loop involving the needle, steel cable, and bone tissue, thereby preventing needle withdrawal. Additionally, slippage of the “8” tension band, a common cause of internal fixation failure, is less likely to occur with the Kirschner wire tension band featuring holes. The specific hole design at the tail effectively secures the steel cable, preventing it from slipping inside the pinhole. The tension exerted by the steel cable in the anchor hole at the distal end of the fracture maintains the steel cable in a tight “8” configuration, which is resistant to relaxation, thus ensuring stability. In contrast, ordinary Kirschner wires merely bend the tail of the nail and embed it into the initial section of the triceps brachii muscle. During functional exercises, when the elbow joint is flexed and extended, these wires are prone to displacement, resulting in loosening of the internal fixation (20).

Perforated Kirschner wires offer certain advantages in minimizing postoperative skin irritation (21). In this study, only one case of skin irritation was reported in the tension band group utilizing perforated Kirschner wires, a significant reduction compared to five cases in the olecranon anatomical plate group and seven cases with ordinary Kirschner wire tension bands. This reduced irritation is attributed to the design of the perforated Kirschner wire, which allows the needle hole to be buried deep within the origin of the triceps muscle along with the steel cable passing through it. This configuration decreases the stimulation of the soft tissue behind the elbow caused by excessive protrusion of the needle tail (22). During the procedure, we observed that when the Kirschner wire is inserted into the medullary cavity but not sufficiently buried, it is advantageous to insert the steel cable into the needle hole first, followed by tapping the needle tail. This technique helps to bury the needle tail deeper into the triceps muscle origin (23). Conversely, if this step is not followed, it may complicate the insertion of the steel cable into the needle hole, necessitating the withdrawal of the needle tail to create adequate operating space for the cable. Furthermore, since one end of the steel cable is threaded through the anchor hole while the other end passes through the needle hole, tightening the cable creates a unified structure that is nearly impossible to slip off. This design limits the sliding and withdrawal of the Kirschner wire, thereby further reducing soft tissue irritation during needle withdrawal. In contrast, the locking plate is relatively thick and rests on the surface of the triceps brachii origin or near the subcutaneous tissue. The irregular shape of the proximal end of the plate can lead to irritation from repeated friction against the surrounding skin during elbow joint extension and rotation (24). Given the delicate nature of the skin over the olecranon of the ulna, local soft tissue complications following plate fixation require careful consideration (25). Despite the differences in skin irritation observed in this study, the long-term joint function outcomes for patients in three groups were satisfactory, with no statistically significant differences. We attributes this to the stability of the internal fixation itself, as both groups emphasized the importance of timely postoperative rehabilitation, resulting in satisfactory joint function.

This study has certain limitations. We did not assign a single surgeon to perform all olecranon internal fixation surgeries; instead, multiple doctors treated their respective patients. This approach may introduce some variability in the test results. However, all participating surgeons are senior physicians with extensive clinical experience, which helps to mitigate the potential impact of individual technical skills on the research outcomes. Secondly, in the research results, we only counted the number of fluoroscopic procedures performed, without specifically recording the ray intensity and total duration of fluoroscopy. This omission represents a limitation of the study. The fluoroscopy utilized is a single type, and there should be a positive correlation between the number of procedures and their duration. Certainly, if data on the specific duration were available, the findings of this study would be more comprehensive.

## 5 Conclusion

The use of perforated Kirschner wire tension band in the treatment of Mayo IIA ulnar olecranon fractures not only shortens the operation time and reduces intraoperative blood loss and the number of fluoroscopy images, but also facilitates the healing of ulnar olecranon fractures with minimal complications. Therefore, appropriate preoperative considerations should be made when selecting a surgical method.

## Data Availability

The raw data supporting the conclusions of this article will be made available by the authors, without undue reservation.
